# Biology, Ecology, and Management of the Invasive *Corythucha arcuata* in Eurasia: A Review

**DOI:** 10.3390/biology15141191

**Published:** 2026-07-19

**Authors:** Cristina Stancă-Moise

**Affiliations:** Department of Agricultural Sciences and Food Engineering, Faculty of Agricultural Sciences, Food Industry and Environmental Protection, “Lucian Blaga” University of Sibiu, 5-7 Ion Ratiu Street, 550003 Sibiu, Romania; cristina.moise@ulbsibiu.ro

**Keywords:** alien forest pests, *Quercus* spp., Integrated Pest Management (IPM), forest biosecurity, climate warming, classical biological control, remote sensing monitoring, phenological plasticity

## Abstract

The oak lace bug, *Corythucha arcuata* (Say, 1832) (Hemiptera: Tingidae), has emerged as an ecologically disruptive invasive pest across Eurasian *Quercus* forests, severely impairing foliar photosynthetic capacity and overall woodland vitality. By systematically reviewing 111 peer-reviewed studies, this comprehensive synthesis examines the biology, geographic invasion dynamics, ecological adaptability, and management of this species. We trace the spatio-temporal expansion of *C. arcuata*, highlighting how sub-zero thermal tolerance, climate-driven polyvoltinism, and phenological plasticity facilitate its rapid establishment across diverse climatic zones. Furthermore, we analyze monitoring frameworks—including MODIS satellite remote-sensing and citizen science networks—while acknowledging both their broad-scale utility and limitations in early detection. Given the severe ecotoxicological constraints of conventional synthetic insecticides, we critically evaluate sustainable Integrated Pest Management (IPM) paradigms. Specifically, we investigate biorational formulations (such as spinosad) alongside promising biological control agents, notably the entomopathogenic fungus *Beauveria pseudobassiana* and the oophagous micro-hymenopteran parasitoid *Erythmelus klopomor*. Ultimately, long-term silvicultural biosecurity requires a multifaceted IPM approach underpinned by continuous geospatial monitoring and rigorous ecological risk assessments prior to classical biocontrol deployments.

## Simple Summary

The oak lace bug (*Corythucha arcuata*) is a severe invasive pest that has rapidly expanded across Eurasia, threatening the vitality and ecological stability of native oak woodlands. This review synthesizes current scientific knowledge regarding this species’ biology, spatiotemporal distribution, and bio-physiological impacts on host trees. We examine its phenology, overwintering resilience, and the environmental drivers facilitating its rapid transcontinental expansion. Furthermore, we analyze existing management strategies, contrasting conventional chemical treatments with biorational alternatives and biological control agents. By identifying critical research gaps, this paper provides guidance for developing robust Integrated Pest Management (IPM) frameworks to safeguard vulnerable forest ecosystems from this ongoing invasion.

## 1. Introduction

On an ecological scale, anthropogenic biological invasions represent a critical threat to the biodiversity, functional architecture, and long-term evolutionary resilience of forest ecosystems. Beyond their profound disruption of native ecological networks, these multi-trophic intrusions generate severe economic externalities; indeed, the aggregate financial liabilities associated with damage mitigation and the containment of invasive alien species across Europe routinely scale into billions of euros annually [1]. Over recent decades, the introduction, establishment, and naturalization rates of non-native phytophagous entomofauna within temperate European landscapes have followed an exponential trajectory. This ecological acceleration is heavily driven by localized climate warming and the dense integration of global trade networks—dynamics robustly captured in comprehensive national inventories, such as those maintained by the Czech Republic, which underscore the acute vulnerability of European temperate woodlands [2]. Within this high-stakes forest biosecurity landscape, the oak lace bug, *Corythucha arcuata* (Say, 1832) (Hemiptera: Tingidae), has emerged as one of the most structurally disruptive foliar pests, prompting severe scientific and silvicultural concern across the Palearctic [3]. Within its native Nearctic range, early research on *C. arcuata* focused primarily on foundational systematics, developmental biology, and morphological micro-structures, such as the ultrastructure of the egg chorion [4]. Early ecological characterizations established that its localized trophic dynamics and vertical micro-distribution within native *Quercus* canopies are strictly modulated by microclimatic and abiotic variables, prominently including solar radiation intensity [5] and host plant moisture deficits [6]. Nonetheless, within these co-evolved habitats, *C. arcuata* maintains a strictly non-epidemic, secondary pest status, with its population densities tightly restricted through homeostatic regulatory feedback loops governed by a complex sympatric guild of natural enemies and region-specific climatic boundaries.

Following its initial Western Palearctic detection in Northern Italy in 2000 [7], *C. arcuata* exhibited a rapid, non-linear spatial expansion across multiple regional borders [8,9,10], quickly advancing from Southern Europe into the core forest landscapes of Anatolia [11,12,13] and the Balkan Peninsula [14,15,16,17]. Subsequently, the insect colonized dense deciduous stands throughout Central Europe; this regional spread was first documented in the Carpathian basin and Romania [18,19,20,21,22,23,24,25], followed by rapid establishment in Hungary and Austria [26,27,28], and a continuous northward progression through Slovakia and the Czech Republic. Concurrently, its geographic range expanded into Eastern Europe, invading broadleaf forests in Poland, Bosnia and Herzegovina [29,30] and expanding into expansive territories in Ukraine [31,32,33]. Over the past two decades, this continuous range expansion has triggered extensive canopy defoliation and severe physiological degradation—impacts widely reported in both natural climax *Quercus* forests [16,17,18,20,21] and heavily anthropized urban green spaces [19,23,24,25,26]. Most recently, active populations have breached Western European biogeographical boundaries [34,35,36,37], confirming the pest’s transition from a localized introduction to a widespread, transcontinental forest management crisis.

The adaptive success of this invasive tingid is fundamentally rooted in its extreme phenological plasticity and robust physiological resilience against otherwise restrictive abiotic constraints. Empirical bioassays demonstrate that this species possesses a sophisticated tolerance to severe sub-zero thermal stress [38,39,40,41,42,43,44], guaranteeing the overwintering survival of a substantial reproductive adult cohort. Furthermore, its dispersal flight kinematics and multigenerational life-cycle dynamics—manifesting as climatically driven facultative polyvoltinism—exhibit a highly significant correlation with regional thermal accumulations and specific bioclimatic indices [45,46,47,48]. The resulting phytopathological toll inflicted upon host *Quercus* species involves the direct mechanical destruction of the photosynthetic apparatus and the induction of complex oxidative biochemical stress cascades [49]. These chronic infestations exert severe cascading repercussions on host fitness, dramatically reducing the viability of the reproductive apparatus and compromising acorn quality [50,51], while downstream impacts degrade the physico-mechanical and technological quality of harvested timber [52]. Compounding this threat, the insect has demonstrated substantial ecological plasticity by adaptively shifting its trophic focus to exploit alternative host plants in heavily anthropized or modified habitats [53].

In response to this transcontinental ecological crisis, the international scientific community has accelerated efforts to design multi-tiered Integrated Pest Management (IPM) strategies. The deployment of advanced territorial monitoring and early detection frameworks has been significantly enhanced by integrating remote-sensing platforms via MODIS satellite sensors [54] alongside the operationalization of collaborative citizen science networks [55]. While localized chemical suppression utilizing synthetic insecticides or biorational formulations, such as the cyclic lactone spinosad, offers transient containment capabilities within forest environments [44,45,46,47,48], the prevailing academic consensus emphasizes a transition toward ecologically sound biological control. Promising avenues for sustainable population suppression include the application of mycoinsecticides formulated from entomopathogenic fungal strains such as *Beauveria pseudobassiana* [56,57], alongside rigorous evaluations of classical biological control through the importation of specialized oophagous micro-hymenopteran parasitoids, most notably *Erythmelus klopomor* [58]. Ultimately, however, the long-term operational success and socio-ecological sustainability of these management programs remain intrinsically dependent upon the baseline awareness, risk perception, and social acceptance of these intervention methodologies by professional stakeholders and local forest-dependent communities [59]. While localized data and regional updates regarding *C. arcuata* are available, the broader academic literature remains fundamentally fragmented and heavily weighted toward isolated faunistic reports. A comprehensive, broad-scale synthesis that bridges the insect’s ecophysiological plasticity with advanced remote-sensing monitoring up to 2026 is conspicuously lacking. This review article directly addresses this critical knowledge gap. By critically analyzing, corroborating, and systematically organizing 26 years of multidisciplinary scientific data, this work establishes an indispensable conceptual and applied framework required to support the long-term conservation, phytosanitary protection, and sustainable management of *Quercus* forest ecosystems across Eurasia.

## 2. Methodology and Literature Retrieval Strategy

### 2.1. Literature Search and Eligibility Criteria

To establish a rigorous, comprehensive, and objective synthesis of scientific data concerning the geographic invasion dynamics, ecophysiology, and integrated management of *Corythucha arcuata* across Eurasia, a systematic literature retrieval protocol was executed. The review encompasses a 26-year temporal window, spanning from the species’ initial official Palearctic detection in 2000 up to June 2026. Primary academic literature was harvested from three major multidisciplinary indexing databases: Web of Science Core Collection (Clarivate Analytics), Scopus (Elsevier), and Google Scholar.

The retrieval matrix utilized structured keyword combinations modulated by Boolean syntax (AND, OR). The primary search equation was formulated as follows:

(“Corythucha arcuata” OR “oak lace bug”) AND (“Eurasia” OR “Europe” OR “biological invasion” OR “Quercus decline” OR “ecophysiology” OR “climate resilience” OR “integrated pest management” OR “biological control” OR “Erythmelus klopomor” OR “Beauveria pseudobassiana”).

Inclusion criteria strictly targeted peer-reviewed journal articles, international conference proceedings, and validated phytosanitary monitoring reports (e.g., EPPO Global Database) providing empirical data regarding the pest’s spatial spread, ecophysiological impacts, or control frameworks.

The screening workflow involved an initial evaluation of titles and abstracts, followed by a thorough full-text eligibility assessment. Strict exclusion criteria were applied to eliminate: publications lacking reproducible empirical data or original observations; gray literature and non-peer-reviewed technical notes lacking verifiable methodologies; and studies focusing exclusively on Nearctic or non-Eurasian populations outside the biogeographic scope of this review.

Although the retrieval strategy predominantly favored English- and Romanian-language literature (potentially introducing a slight linguistic bias), systematic efforts were deployed to integrate pivotal faunistic and forest health studies from other Palearctic regions via international databases and verified abstract translations. This dual approach guarantees that the synthesis captures both regional Eurasian dynamics and localized silvicultural case studies.

Following a multi-tiered, PRISMA-adapted screening protocol that included deduplication, title and abstract screening, and full-text validation, a final corpus of 111 scientific documents was selected, critically analyzed, and integrated into this review.

### 2.2. Geospatial Mapping and Spatial Vector Reconstruction

**Figure 1:**
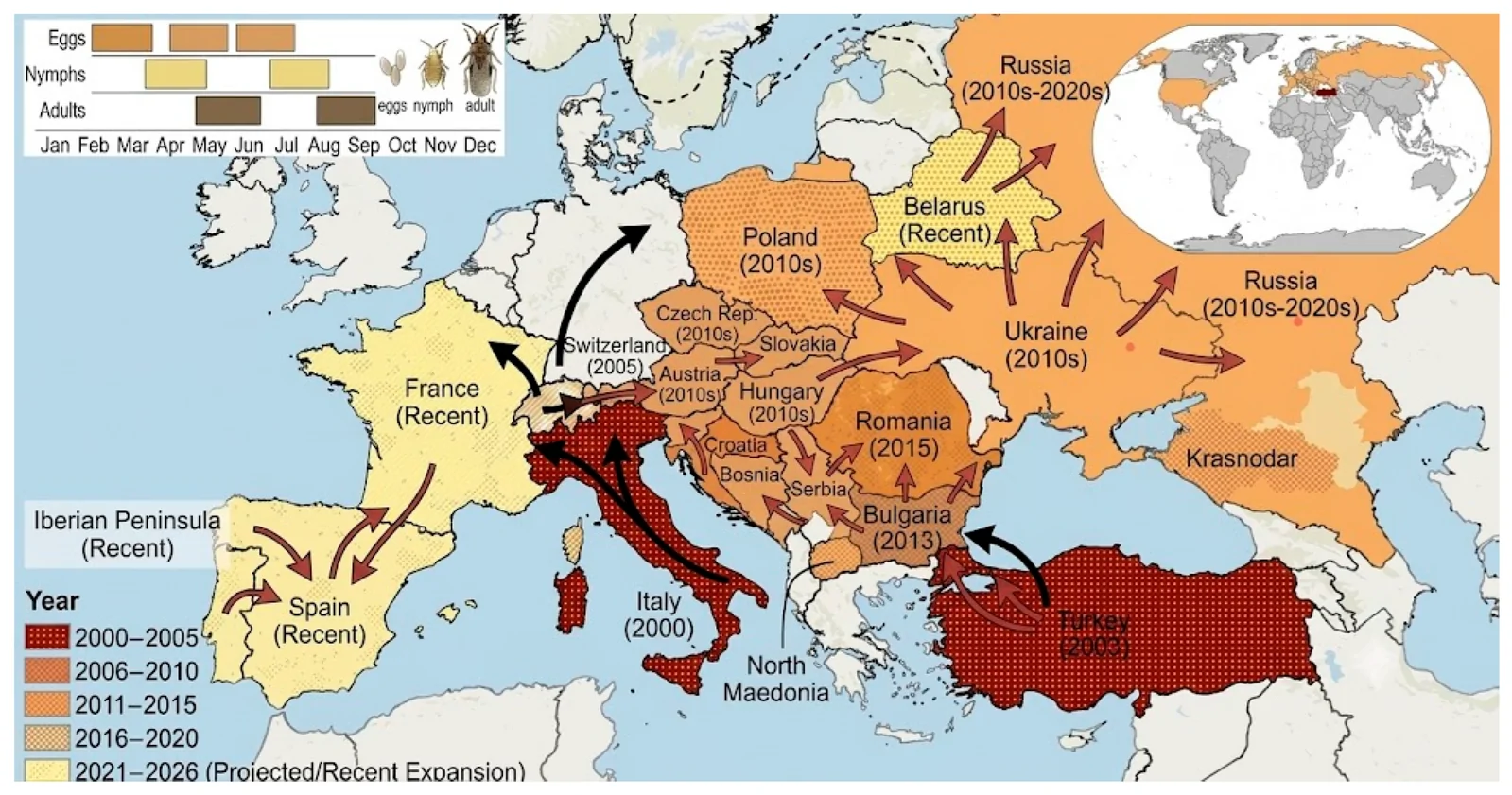
Chronological expansion and comprehensive distribution map of the oak lace bug (*Corythucha arcuata*) in Eurasia from 2000 to 2026. Note: Textured patterns applied within administrative country borders represent localized, patchy presence zones rather than a continuous, border-to-border geographic distribution. Shaded regions indicate established, self-sustaining territorial records categorized sequentially by colonization periods. Black vectors delineate primary historical invasion pathways and long-distance jump dispersals reconstructed from earliest chronological hubs; red vectors represent secondary transboundary, localized, and contiguous expansions through European forest corridors.

To visualize the transcontinental expansion and contemporary Palearctic distribution of *C. arcuata* (Figure 1), a chronological geospatial database was compiled using verified national and regional first-detection records extracted from the synthesized literature. Geographic Information System (GIS) software (QGIS, version 3.34; QGIS.org) was employed to map invaded territories and reconstruct historical migration corridors.

Spatial attribution and cartographic symbology were standardized according to the following criteria: Territorial shading (Presence status). A fine stippling (textured dot) pattern was applied within countries or administrative sub-regions where self-sustaining, reproducing populations and associated structural canopy defoliation have been officially confirmed by national phytosanitary authorities.

It must be explicitly emphasized that country-level shading reflects administrative presence and historical colonization periods only; it does not imply that the species continuously occupies the entire national territory of each country within its administrative borders.

In reality, actual populations remain strictly localized within specific Quercus forest basins, riparian corridors, and favorable urban green infrastructure.

Unshaded (white) territories represent areas with no confirmed naturalization or where only transient, non-established interceptions have occurred.

## 3. Taxonomy, Systematics, and Morphology

### 3.1. Taxonomic Status and Evolutionary Context

The oak lace bug, *Corythucha arcuata* (Say, 1832), is systematically classified within the order Hemiptera, suborder Heteroptera, family Tingidae, and subfamily Tinginae. Members of the family Tingidae, commonly known as “lace bugs” due to the reticulated, finely fenestrated, and hyaline architecture of the adult exoskeleton, constitute an exclusively phytophagous insect group characterized by a pronounced trophic specificity (oligophagy or monophagy). The genus *Corythucha* Stål, 1873, exhibits a remarkable center of diversification in the Nearctic region, encompassing a large number of species native to North America. Early efforts toward a rigorous taxonomic revision and classification of this genus commenced in the early 20th century, focusing on the development of dichotomous keys based on the morphological characters of the pronotum and the configuration of the hemelytral cells [60]. The family Tingidae and the genus *Corythucha* comprise phytophagous taxa with a vast geographical distribution and remarkable ecological plasticity; extensive faunistic studies have confirmed the adaptability of this group to highly diverse habitats, ranging from temperate forest phytocoenoses to the arid and semi-arid regions of the Middle East, such as Iran [61].

The nomenclature and systematics of the family were substantially consolidated through the publication of the world catalog of tingids, a comprehensive taxonomic study that clarified historical synonymies, early geographic distribution, and the phyletic relationships of *C. arcuata* in relation to other members of the genus [62]. In the contemporary period, driven by advancements in cladistic analysis techniques and molecular phylogeny, the taxonomic position of this species and the dynamics of evolutionary networks within the Tingidae family have undergone periodic re-evaluations, providing an integrated perspective on the evolution of these partially xerophilous insects and their associated species complexes [62].

### 3.2. Morphological Characteristics and Differential Diagnosis

Adults of *C. arcuata* are small, with body length typically ranging between 3.0 and 3.5 mm. Anatomically, the species is distinguished by an exceptionally developed pronotum, equipped anteriorly with a median vesicle protecting the cephalic capsule, and widened lateral expansions (paranota); all these structures display a reticulated, transparent texture composed of microscopic areoles (cells). However, reliable taxonomic identification is not restricted solely to the imago stage. High-resolution ultrastructural investigations performed via scanning electron microscopy (SEM) have elucidated fine morphological details of critical importance regarding the chorion of the *C. arcuata* egg, highlighting the structural complexity of the operculum and the specific micro-sculpture of the outer surface [63]. These chorionic micro-characters possess high diagnostic value in population dynamics studies, allowing early identification of the species from the embryonic stage and its differentiation from other native or alien tingids.

In its newly invaded range in Europe, a major diagnostic impediment is the partial overlap of the ecological niche and the high morphological similarity with another related invasive phytophagous pest—the sycamore lace bug, *Corythucha ciliata* (Say, 1832). Nevertheless, recent comparative analyses of external morphological characters have provided rigorous criteria for the differential diagnosis of the two taxa [64]. Unlike *C. ciliata*, which has a predominantly milky-white and immaculate body with a single small dark macula on the pronotal vesicle, *C. arcuata* is clearly distinguished by well-defined, brown transverse bands arranged in an arcuate pattern at the base and in the apical region of the hemelytra. Furthermore, definitive morphological discrimination relies on the shape and convexity of the pronotal hood, as well as on the number of rows of areoles defining the outer margins of the paranota [64].

## 4. Spatiotemporal Invasion Dynamics and Geographic Expansion

The spatiotemporal progression of *Corythucha arcuata* across the Western Palearctic bypasses classical, linear biogeographical invasion models. Instead, its colonization of Eurasia is characterized by a highly dynamic, stratified diffusion process—a synergistic combination of localized, short-range active flight (stepping-stone diffusion) and long-distance, human-mediated jump dispersal via international trade and transportation corridors. Rather than exhibiting a uniform radial expansion, regional dynamics reveal distinct geographical waves that rapidly transformed a localized Mediterranean introduction into a synchronized, transcontinental forest health crisis.

### 4.1. The Mediterranean Introduction and Balkan Range Expansion

Following its primary introduction into Northern Italy [7,8], *C. arcuata* established a rapid secondary expansion pathway along the eastern Mediterranean basin and the Balkan Peninsula. In Anatolian oak ecosystems, the pest encountered favorable thermal regimes that accelerated population growth from isolated foci into extensive, chronic forest outbreaks [65]. Climatic analysis indicates that these established populations in Asia Minor acted as permanent, high-density sources for subsequent transboundary dispersal vectors across the Bosporus [11,12,13].

Using these eastern populations as a demographic springboard, this tingid systematically penetrated the Balkan corridor [14,15,16]. The exceptionally rapid establishment within these territories was facilitated by a critical host-phenology synchrony, allowing emerging nymphs to exploit early-season foliar resources [66]. Within the lowland forest areas of Croatia, particularly inside the contiguous, high-value *Quercus robur* stands of the Spačva forest basin, the biotic pressure exerted by *C. arcuata* reached catastrophic thresholds [17].

Spatial modeling confirms that the high connectivity of oak stands within these landscapes significantly accelerated the pest’s localized dispersal rate and reproductive success [67,68,69,70]. The intersection of dense host-plant availability and optimal microclimates fueled massive population spikes, resulting in widespread canopy defoliation and initiating a rapid centrifugal expansion that spilled into neighboring forest basins across Serbia Bosnia and Herzegovina, Montenegro, North Macedonia [30,71,72,73]. These chronic, multi-annual defoliation events were directly correlated with the increased susceptibility of weakened hosts to secondary bark beetle infestations and opportunistic buprestid wood-borers [68]. Furthermore, the deployment of standardized, long-term monitoring protocols across these Balkan hubs has proven essential in quantifying the progressive decline of forest canopy vigor [69].

### 4.2. Continental Saturation: The Central and Eastern European Frontiers

As the Balkan and Anatolian population centers reached demographic saturation, the geographic range expansion of *C. arcuata* shifted toward higher latitudes and longitudinal extremes of the European continent. This phase of the invasion successfully refuted early bioclimatic models that predicted strict northern and eastern distribution limits due to lethal winter thermal constraints.

Within this continental dynamic, Romania represented a pivotal transition zone. Following its first official detection in the western part of the country [74], the species rapidly colonized national forest lands, establishing a high-density endemic plateau [18,19,20,21].

Research across varying ecoclimatic gradients has demonstrated that peak diurnal flight activity correlates strongly with specific micrometeorological thresholds [43,44]. Furthermore, this colonization has induced chronic chlorotic degradation not only in natural climax oak ecosystems but also within urban and peri-urban dendrological networks, effectively utilizing urban infrastructure as dispersal corridors [22,23,24,25,75,76,77,78,79,80].

The Western Alpine valleys of Switzerland were breached early in the invasion sequence [9], providing early empirical evidence of the insect’s latent capacity to adapt to alpine-temperate transition zones and demonstrating a high ecological plasticity outside its Nearctic climate envelope [10]. This orographic transition catalyzed a highly synchronized Central European expansion wave that consolidated the pest’s presence across Hungary, Austria, Slovakia, and the Czech Republic [27,81]. In Hungary, researchers documented the accelerated degradation of oak health under the combined pressure of tingid attacks and secondary environmental stressors [26,77]. Host adaptation to Central European winter regimes was further validated through ecophysiological studies on the ecological success and supercooling capacity of overwintering adult forms [40,41,42]. Subsequently, the insect penetrated Austria [28,78], generating phytosanitary risk analyses focused on the vulnerability of Central European silvicultural resources [79], while synchronously expanding into Slovakia [27,80] necessitating an immediate revision of national forest protection and quarantine frameworks.

Mirroring this upward trajectory, an ascending invasive vector pushed northward into Poland [29,82], actively expanding the pest’s septentrional climate envelope and challenging the limiting ecological capacities of the species at the northern boundary of the native *Quercus* range [30]. Further east, *C. arcuata* successfully adapted to severe continental constraints, causing massive, high-density outbreaks across the Krasnodar region, the southern forest basins of the Russian Federation [64,83], and deep into the vulnerable forest-steppe ecosystems of Ukraine [31,32,33,84], displaying an extraordinary cold-hardiness during its overwintering adult stage. The eastward expansion of the pest continued uninterrupted across the Eurasian landmass, favored by high landscape connectivity, the lack of major geographical barriers, and a dense international transport network, with recent verifications in the Kharkiv region confirming its capacity to rapidly exploit new anthropogenic corridors [31,84]. Further north, in Belarus, early interceptions of the species have been recorded in the southern regions, though its precise distribution limits and population densities currently require more extensive monitoring.

### 4.3. Western European Expansion and Continental Consolidation

The final phase of this transcontinental encirclement occurred during the last decade, marking the closing of the European invasive circle through the systematic penetration of Western Europe and the Iberian Peninsula—territories historically deemed protected by geographic isolation and topographic barriers. Biologically active, reproducing populations were officially validated across France, Spain, and Portugal [34,35,36,85,86,87,88,89,90,91].

In France, the species was initially intercepted through national epidemiological surveillance networks, which detected localized point-source introductions [86]. Despite the immediate enforcement of statutory quarantine protocols, the exceptional passive and human-mediated dispersal capacity of this heteropteran bypassed regional containment lines, establishing reproducing populations across major French forest basins and registering a continuous spatial expansion across an increasing number of administrative departments [87]. Concurrently, the Iberian Peninsula [88] was breached through localized outbreaks initially clustered along strategic border zones and the highly humid Atlantic facade. The first official records spanning northern Spain and Portugal [36,88,89] were immediately followed by a high-density colonization of the northeastern regions and Catalonia, where its highly volatile population dynamics and multivoltine life cycles have been closely monitored [34,35,36,88,89,90,91]. The chronological baseline of this transcontinental range expansion is systematically compiled in Table 1.

The colonization of these western frontiers—frequently mediated by long-distance anthropogenic hitchhiking vectors along major trade corridors—directly threatens ancient, ecologically vital Atlantic and Mediterranean oak associations. State-of-the-art biogeographical analyses and ecological niche modeling (ENM) demonstrate that *C. arcuata* is actively occupying a significant portion of its potential ecological niche in Western Europe. Fueled by rising summer thermal regimes, this pest directly threatens the structural and functional stability of native Atlantic and Mediterranean oak associations [90,91], establishing a state of chronic phytosanitary emergency across the southwestern extremities of the continent (Figure 1).

## 5. Ecology, Phenology, and Climate Resilience

The successful establishment of *Corythucha arcuata* in the newly colonized territories of Eurasia is driven by a synergy between high ecophysiological plasticity and a lack of top-down population control. The accelerated proliferation of this species can be partially attributed to the Enemy Release Hypothesis; native Palearctic predators and parasitoids have not yet adapted to effectively regulate these novel populations, allowing *C. arcuata* to exploit host resources with minimal biological resistance. Furthermore, this absence of coevolved natural enemies is complemented by the insect’s capacity to modulate its developmental cycles and metabolic responses in accordance with local macro- and microclimatic variations. This dual advantage enables the species to optimize its overwintering survival and maximize its reproductive output during the vegetative period.

### 5.1. Phenological Plasticity and Life Cycle Voltinism

**Figure 2:**
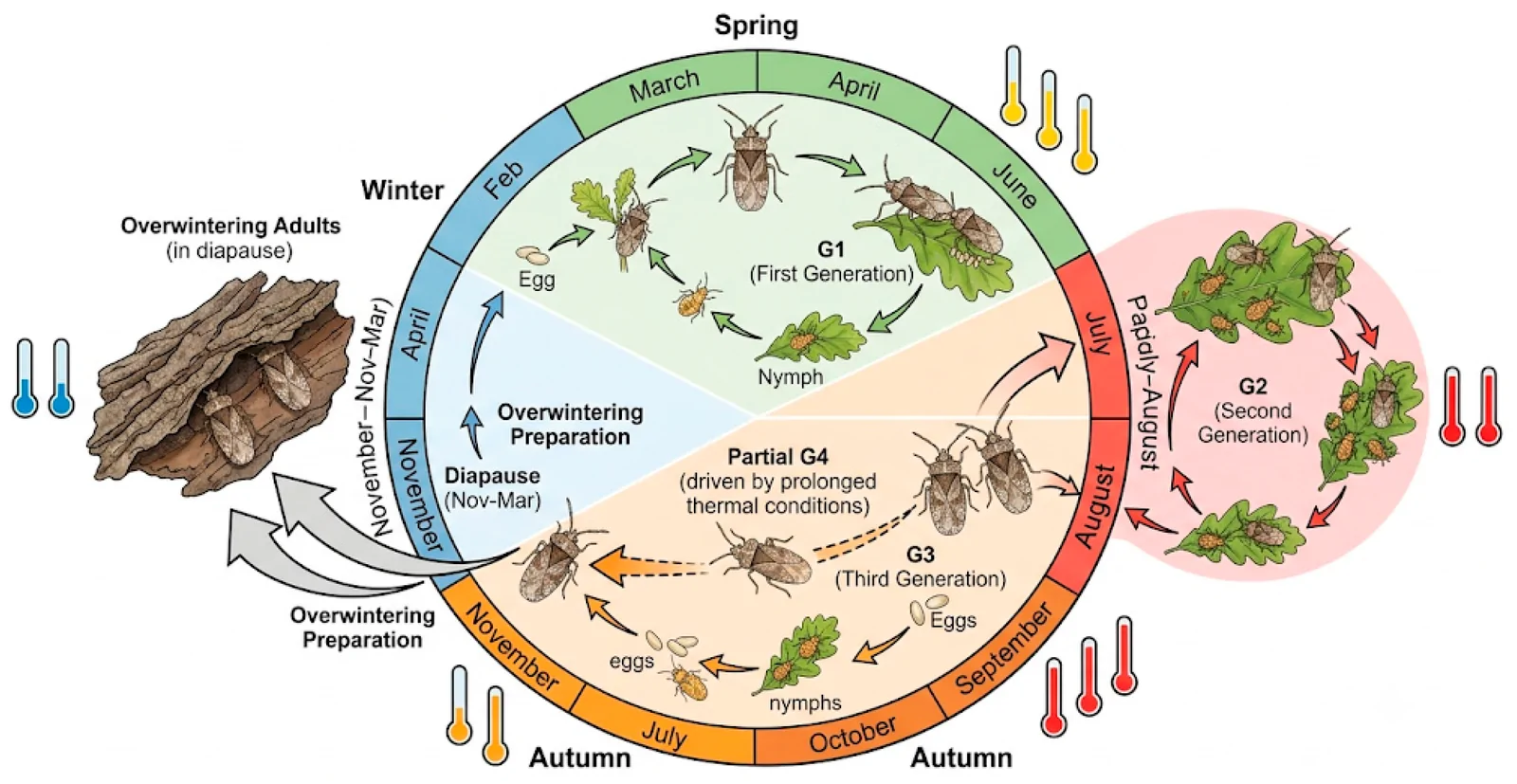
Schematic representation of the temperature-driven polyvoltine life cycle of *Corythucha arcuata* in Europe, illustrating the succession of generations (G1–G4), overwintering dynamics, and the synchronization with seasonal thermal fluctuations. Circular colored arrows indicate the development and succession of active generations (green/red for G1–G2, orange for G3–G4). Large grey arrows show the migration of adults toward shelter under tree bark (overwintering preparation), dashed orange arrows indicate late-season adults transitioning to the overwintering pool, and the inner blue arrow represents the progression of the winter diapause.

Within its newly acquired European range, *C. arcuata* has transitioned from a relatively stable voltinism (monovoltine or partially bivoltine in colder native regions) to a pronounced polyvoltine character. This phenological shift is closely linked to the accumulation of thermal units required for the completion of the egg, nymphal, and adult stages. Long-term studies conducted by Stancă-Moise et al. have contributed significantly to deciphering these mechanisms [22,23,24,25,92,93]. Their research, spanning several consecutive years, precisely mapped the pest’s phenological calendar, demonstrating the consistent occurrence of two to three complete generations per year, with a partial fourth generation frequently emerging during years with prolonged autumns (Figure 2). This accelerated development results in a pronounced overlap of life stages within the canopy, significantly complicating the identification of optimal intervention windows for integrated pest management [25].

The dynamics of local dispersal and the diurnal flight behavior of adults exhibit strong meteorological influence. Bălăcenoiu et al. [43,44] investigated the complex interaction between micrometeorological factors and flight intensity in oak forests (*Quercus* stands), demonstrating that peak diurnal flight activity is directly triggered by specific thresholds of air temperature and global solar radiation, whereas high relative humidity acts as a major inhibitory factor. This ecoclimatic synchronization optimizes mate-seeking behavior and the colonization of new host trees.

The influence of temperature on the intrinsic rates of population increase was thoroughly investigated by Knor [94], whose experimental models established the lower developmental thresholds and the sums of effective temperature required for each nymphal stage. It was demonstrated that the microclimate within the canopy, particularly at the level of sun-exposed leaves, reaches temperatures considerably higher than those recorded by standard meteorological stations, massively accelerating the insect’s metabolism [94]. Complementarily, the seasonal abundance curves evaluated by Stattler [95] highlight that population densities reach a critical peak in mid-to-late summer (July–August). This demographic apogee temporally coincides with periods of maximum host tree water stress, synergistically accelerating the degradation of the oak canopy through the destruction of palisade cells [95] (Figure 2).

### 5.2. Cold Tolerance and Overwintering

Survival during the cold season represents another critical pillar of the invasive success of *Corythucha arcuata*, enabling this species to withstand the rigors of continental winters in Central and Eastern Europe. Overwintering occurs exclusively in the adult (imago) stage, with individuals aggregating in dense colonies under the rhytidome (exfoliating bark) of oak trees, within deep trunk crevices, or beneath the leaf litter.

Research conducted in the Pannonian region has demonstrated that overwintering success is exceptionally high, with adult survival rates frequently exceeding 70–80% under standard winter conditions [40,41,42]. This success is partially due to the insect’s capacity to enter a state of deep facultative diapause, which is bioenergetically modulated based on resources accumulated during autumn. This aspect was further confirmed through the use of predictive models based on atmospheric heat content and pre-winter thermal accumulations [45,46,47]. The authors demonstrated that the optimal accumulation of thermal units prior to the onset of cold conditions induces essential biochemical modifications in the fat body of adults, physiologically preparing them for survival at extreme sub-zero temperatures [45,46,47].

The energetic preparation of this insect depends directly on nutrient assimilation from the host foliage prior to senescence, a phase during which the accumulation of soluble carbohydrates plays a crucial role in altering food quality and, subsequently, depressing the pest’s supercooling point; the quantification of these essential carbohydrate compounds in plant tissues is frequently achieved via optimized spectrophotometric methods [96].

The fine physiological mechanisms underlying frost resistance have been elucidated through ecophysiological studies [37,38]. Determination of the supercooling point (SCP) has revealed that *C. arcuata* adopts a freeze-avoidance strategy, avoiding tissue water crystallization through the accumulation of endogenous polyol cryoprotectants (such as glycerol and sorbitol) in the hemolymph [38]. Nevertheless, overwintering mortality can increase significantly in the event of abrupt thermal fluctuations or prolonged episodes of severe frost lacking a protective snow cover [37]. However, the current trend of global climate warming and the increasing frequency of mild winters in Europe are likely to reduce this hibernal mortality, although local microclimatic variations dictate that such effects should not be broadly generalized across all European regions.

Despite these identified mechanisms, the exact limits of prolonged cold exposure and the energetic costs of overwintering on adult cohorts remain partially undocumented and warrant further investigation.

### 5.3. Population Genetics and Adaptive Diversity

Theoretically, the rapid colonization of a new continent by an exotic species is often accompanied by a severe founder effect and a drastic reduction in genetic diversity (genetic bottleneck), phenomena that theoretically limit the adaptability and expansion of invasive populations. However, in the case of *C. arcuata*, these genetic constraints appear to have been successfully overcome.

The analysis of the genetic structure of invasive Eurasian populations, conducted by Besedina and Kil [47], provided valuable insights into the molecular basis of this resilience. Utilizing molecular markers (such as the sequencing of the mitochondrial cytochrome oxidase I gene COI), their research revealed a surprisingly robust genetic diversity within Eastern European outbreaks [47]. This molecular signature suggests that the invasion of Europe was not the result of a single, isolated introduction event, but rather the consequence of multiple and repeated introductions from distinct geographical sources within its native Nearctic range. This constant influx of new genetic material counteracted the deleterious effects of inbreeding and maintained a polymorphic adaptive gene pool, providing the necessary genetic diversity to facilitate rapid selection and high phenotypic plasticity in response to novel selective pressures within Palearctic forest ecosystems [47]. However, conclusions regarding multiple introduction pathways based largely on single-locus COI data should be interpreted with caution. Future phylogeographic studies incorporating genome-wide markers are necessary to fully resolve the invasion pathways and adaptive genetic architecture of these populations.

## 6. Host Plants and Bio-Physiological Impact

The capacity of the species *Corythucha arcuata* to inflict major damage within its newly acquired European habitats resides in the complex interaction it establishes with its host trees. Although initially described as a species with a relatively narrow trophic spectrum restricted to the genus *Quercus*, monitoring within invaded ranges has revealed feeding plasticity and a far more profound ecophysiological impact than previously anticipated.

### 6.1. Physiological Impacts on Quercus Species and Canopy Degradation

Within European forest ecosystems, native oak species represent the primary and preferential hosts of the pest, upon which it can complete its entire developmental cycle. The mechanism of attack consists of piercing the abaxial epidermis of the leaves and extracting intracellular contents, a process that leads to the destruction of the palisade tissue and chloroplasts. This feeding behavior disrupts the physiological balance of the tree. Ecophysiological research conducted by Nikolić et al. [48] has demonstrated that severe infestations lead to a reduction in stomatal conductance and photosynthetic assimilation rate, thereby affecting water balance and the capacity of the trees to accumulate biomass. Specifically, infestations can reduce the photosynthetic assimilation rate by up to 58%, depending on the extent of foliar damage and the targeted *Quercus* species [23].

The impairment of leaf functionality can trigger subsequent effects across the biocenosis. A consequence of this resource deficit is the potential alteration of the trees’ reproductive output. Franjević et al. [50] documented a decrease in the quality and viability of seeds (acorns) in heavily infested pedunculate oak stands, an aspect that may affect natural regeneration, though the long-term implications for forest dynamics require continuous, long-term monitoring. Furthermore, repeated attacks over several growing seasons can negatively influence dendrometric indices and timber quality in certain species. For instance, a study conducted specifically on *Quercus frainetto* highlighted structural modifications in annual growth rings and a decline in the technological properties of wood under the pressure of continuous attacks [51].

The manifestation of this impact also depends on the architecture and composition of the forest ecosystem. Evaluating the interaction between this pest and stand structure, Hoch et al. [79] revealed that infestation intensity and the degree of defoliation are significantly higher in pure oak stands compared to mixed stands, underscoring the role of tree species diversity in mitigating attack pressure. This phenomenon is further influenced by general herbivory patterns, where biotic stress induced by *C. arcuata* often overlaps with other abiotic factors (such as drought) or attacks by other defoliators, contributing to a complex forest decline syndrome [97].

The biotic pressure exerted by *C. arcuata* is also notable in humid habitats, such as floodplains and mixed broadleaf forests. These ecosystems, characterized by specific plant associations where oak species play a structural role—as described in river basins such as the Mirna River in Croatia—are susceptible to these outbreaks; chronic defoliation may affect the vigor of mature trees and influence the floristic composition of the understory [98].

### 6.2. Alternative Host Exploitation and Spread in Urban Environments

Although oaks remain the preferred target, immense population pressure and the depletion of primary trophic resources during demographic peaks trigger a broadening of the host spectrum. From the early stages of the European invasion, Bernardinelli [53] investigated the polyphagous potential of the species, demonstrating through laboratory tests and field observations that nymphs and adults can feed and survive on a variety of other deciduous species. This “opportunistic polyphagy” was subsequently confirmed by Kovacs et al. [99], who reported active infestations and the development of *C. arcuata* generations on sweet chestnut (*Castanea sativa*), a species of major economic and ecological importance.

This trophic flexibility facilitated the massive infiltration of this pest into anthropogenic ecosystems. Urban green spaces, street tree alignments, and botanical gardens offer warmer microclimates (“urban heat islands”) that favor the development of the oak lace bug. In Romania, this phenomenon has been extensively documented. The first reports of its presence on multiple alternative hosts were recorded in the Macea Botanical Garden, where the insect was collected from various ornamental and forest woody species [100].

Invasion dynamics within urban environments have been investigated in detail across large urban agglomerations, such as the municipality of Bucharest. Here, Bălăcenoiu et al. [21] mapped the presence of the pest in major parks and recreational areas, reporting critical infestation levels that severely affect the aesthetic value of woody plants. The expansion of the host plant spectrum and adaptation to the diversified urban landscape were also corroborated by research conducted by Ciceoi et al. [101,102], which highlighted the visual and ecological impact of the insect in urban tree alignments. More recently, extensive monitoring carried out by Stancă-Moise [22] in urban and peri-urban areas confirmed that *C. arcuata* utilizes the ecological infrastructure of cities not only as a secondary habitat but also as genuine “dispersal corridors” to facilitate long-distance spread.

## 7. Monitoring, Control, and Integrated Management

Efficient management of invasive populations of *Corythucha arcuata* in the Palearctic region represents a major challenge for forestry, as traditional forest protection methods have often proven insufficiently adapted to the regional scale of this invasion. Successful management necessitates a shift from reactive strategies to a proactive Integrated Pest Management (IPM) framework, coupling modern monitoring technologies, targeted biocontrol interventions, and social acceptance of the applied measures.

### 7.1. Remote Sensing and Citizen Science for Spatial Monitoring

Early identification of outbreaks and mapping of dispersal dynamics are essential for mitigating the risk of massive infestations. In recent decades, remote sensing techniques have revolutionized forest health monitoring. Berta et al. [54] demonstrated the efficacy of utilizing high-temporal-resolution MODIS satellite imagery to detect specific spectral signatures associated with foliar discoloration induced by lace bug feeding in oak stands. This approach enables the scanning of vast forested areas and the identification of zones undergoing physiological decline before damage becomes irreversible at the stand level.

To enhance the accuracy of predictive models, satellite data have been correlated with environmental variables. Kern et al. [103] developed complex algorithms that combine vegetation indices obtained via MODIS satellites with high-resolution meteorological data (temperature, water deficit). These models have proven capable of anticipating the pest’s seasonal dynamics and localizing forest areas with the highest ecoclimatic vulnerability. In parallel with satellite technologies, opportunistic data collection through citizen science platforms has emerged as a valuable complementary tool. De Groot et al. [55] highlighted how involving the general public and volunteers in reporting the presence of *C. arcuata* via mobile applications provides researchers with precise spatiotemporal data, which are crucial for the rapid detection of new invasion fronts at the boundaries of biogeographic ranges.

### 7.2. Synthetic and Biorational Chemical Control

Although the application of insecticides in forest ecosystems is strictly regulated due to ecotoxicological considerations, chemical intervention remains a necessary last resort in cases of severe population explosions that threaten stand survival. Bălăcenoiu et al. [43] evaluated the efficacy of various chemical substances applied in oak forests, establishing protocols to optimize ground and aerial treatments while minimizing impacts on non-target fauna. Synchronizing treatments with windows of maximum vulnerability within the insect’s phenology (such as the emergence of the first spring nymphs) has proven crucial for reducing population densities.

The comparative evaluation of different insecticide classes was further investigated by Drekić et al. [104], who tested the biological efficacy of several synthetic compounds under controlled and field conditions. Their research showed that neonicotinoids and pyrethroids deliver high short-term mortality rates, yet the risk of inducing resistance and adversely affecting beneficial entomofauna persists [104]. Consequently, scientific interest rapidly shifted toward ecological solutions and biorational preparations. Besedina et al. [46] tested the use of plant-derived insecticides and insect growth regulators, obtaining promising results in reducing nymphal viability while maintaining a significantly more favorable ecotoxicological profile. Within this bio-insecticide context, Ghergheles et al. [75] investigated the specific impact of Spinosad (an insecticide derived from the fermentation of the bacterium *Saccharopolyspora spinosa*), demonstrating that it exhibits remarkable efficacy against both adults and nymphs of *C. arcuata*, thereby representing an effective biorational option for integration into management programs for valuable oak stands or urban green spaces.

### 7.3. Biological Control: Entomopathogenic Fungi and Oophagous Parasitoids

Classical or augmentative biological control offers a key component of long-term sustainable management for restoring ecological balance in invaded European forests.

**Entomopathogenic Fungi**: Native pathogenic microorganisms represent a primary biological line of defense. Kovač et al. [56,57] conducted extensive screenings to identify virulent fungal strains capable of infecting the oak lace bug. Their research focused on the species *Beauveria pseudobassiana*, demonstrating through laboratory tests and field bioassays that the isolation and application of this entomopathogenic fungus leads to high mortality rates among *C. arcuata* populations via cuticle penetration and hyphal multiplication within the hemocoel. These fungi offer the additional advantage of surviving within overwintering microhabitats (beneath the rhytidome), thereby limiting the number of adults that resume activity in the spring [57].

Although *C. arcuata* is an exotic species in Europe that has escaped its native natural enemy complex, recent years have witnessed incipient adaptation of local entomophagous fauna to this novel food source. Accordingly, cases of natural egg and nymph parasitism have been recorded in Southeastern European regions; for instance, research in Serbia has highlighted the limited biological control potential of certain indigenous species that are beginning to exert an effect on oak lace bug populations [105].

**Parasitoids:** The most promising avenue for classical biological control involves the use of oophagous parasitoids. Within its native North American range, the population dynamics of *C. arcuata* are naturally regulated by the parasitoid wasp *Erythmelus klopomor* (Hymenoptera: Mymaridae), which has been documented as a highly effective biological control agent in regions such as Missouri, significantly reducing the viability of this pest’s egg clutches [106]. The bio-ecological characteristics and host specificity of *E. klopomor* were initially described in Nearctic ecosystems by Puttler et al. [106].

Given the ecological crisis in Europe, scientific efforts have been initiated to evaluate the potential introduction of this biocontrol agent onto the European continent. Paulin et al. [58] established the procedural steps and ecological risk analyses required for implementing classical biological control with *E. klopomor* in Europe. Preliminary specificity studies indicate that this parasitoid exhibits high fidelity toward the genus *Corythucha*, thereby minimizing the risk of adverse impacts on native heteropteran species, paving the way for controlled releases in the near future [58] (Table 2).

### 7.4. Socio-Ecological Dimensions of Integrated Pest Management

The implementation of an integrated management program extends beyond the boundaries of strictly biological research, necessitating the adaptation of national and transboundary forestry policies. Regional strategies developed in countries with advanced forestry sectors emphasize the importance of combining silvicultural methods (such as promoting mixed stands and reducing density through adapted thinning operations) with digital monitoring and targeted biological interventions [107]. These management frameworks highlight that pest eradication is no longer a realistic objective, with the current goal being the maintenance of populations below the economic injury level [108]. Holistic analyses suggest that intervention decisions must be grounded in rigorous landscape-level ecological risk assessments [109,110,111].

An often overlooked yet vital aspect of the success of any IPM strategy is the human and social dimension of invasive species management. Bălăcenoiu et al. [59] conducted an extensive European-level sociological study, investigating the perception, knowledge levels, and willingness to act of field forestry personnel and ordinary citizens. The results revealed a significant discrepancy between awareness of attack severity and acceptance of control methods (particularly chemical ones in the proximity of inhabited areas). This study demonstrated that the long-term efficacy of control and oak forest conservation measures directly depends on public information campaigns, decision-making transparency, and engaging local communities in participatory monitoring programs, thereby securing the social support necessary for funding and applying modern biocontrol techniques [59] (Table 3).

## 8. Conclusions

The transcontinental expansion of *Corythucha arcuata* across Eurasia represents a critical ecological perturbation threatening the stability of Palearctic *Quercus* forest ecosystems. Driven by extreme phenological plasticity, climatically modulated polyvoltinism, and a robust adaptive capacity to diverse climatic regimes, this tingid has successfully established breeding populations across extensive geographic frontiers. Its ability to overwinter efficiently under severe thermal stress and exploit broad environmental gradients has been central to its rapid naturalization in newly invaded territories.

As this species continues to consolidate its presence in saturated zones and advance into new European frontiers—most notably the Iberian Peninsula—continuous territorial monitoring remains indispensable. Advanced geospatial detection tools, such as MODIS satellite remote-sensing platforms and collaborative citizen science networks, provide invaluable broad-scale distribution data; however, their operational limitations regarding early localized detection and inherent identification biases must be explicitly factored into biosecurity planning.

In terms of population management, reliance on conventional chemical treatments is severely constrained by ecotoxicological hazards, economic costs, and logistical barriers within natural forest landscapes. Consequently, transitioning to Integrated Pest Management (IPM) frameworks represents the only viable silvicultural strategy. While biologically based biological control—utilizing natural enemies such as entomopathogenic fungi and specialized oophagous parasitoids—holds substantial promise for long-term population suppression, any importation or augmentative release of biological agents must be strictly preceded by rigorous ecological risk assessments to preclude unintended trophic disruptions to non-target fauna and native food webs.

Ultimately, future research paradigms must prioritize the acquisition of quantitative, longitudinal data evaluating the chronic impacts of *C. arcuata* on host photosynthetic efficiency, acorn viability, and natural forest regeneration. Expanding the empirical evidence base regarding native predator interactions and refining standardized, ecologically sustainable phytosanitary protocols will be crucial for safeguarding the evolutionary resilience of Eurasian oak woodlands under an accelerating climate change trajectory.

## Figures and Tables

**Table 1:** Chronological sequence and geographic expansion milestones of *Corythucha arcuata* in Eurasia (2000–2026).

Regional Wave	Country/Region	First Record	Primary Dispersal Dynamics and Ecological Context	Key References
I. Mediterranean and Balkan Core	Italy (Lombardy)	2000	Primary Western Palearctic gateway; rapid Po Valley radial diffusion.	[7,8,65,66,67,68,69]
	Turkey	2003	Anatolian scaling; permanent transboundary source-sink across the Bosporus.	[11,12,13]
	Bulgaria and Croatia	2013	Penetration of the Balkan corridor; host-phenology synchrony.	[14,15,16,17]
	Serbia, Bosnia and Herzegovina, Montenegro, North Macedonia	2010s	Centrifugal spillover driven by contiguous lowland oak matrices.	[30,71,72,73]
II. Continental and Eastern Frontiers	Switzerland (Ticino)	2005	Early Alpine–temperate transition zone adaptation.	[9,66]
	Romania	2015	Rapid saturation of Carpathian climax forests and urban green spaces.	[18,19,20,21,22,23,24,25,74]
	Hungary, Austria, Slovakia, Czech Republic	2010s	Central European expansion wave; high overwintering supercooling capacity.	[26,27,28,40,41,42,78,79,80,81]
	Poland	2010s	Septentrional range expansion; testing of northern climatic boundaries.	[29,82]
	Ukraine and the Russian Federation	2010s–2020s	Massive outbreaks in forest-steppes; extreme continental cold-hardiness.	[31,32,33,64,83,84]
III. Western Frontiers	France	2020s	Point-source introductions bypassed containment via transport networks.	[85,86,87]
	Spain and Portugal	2020s	Breach of the Iberian Peninsula and humid Atlantic facade.	[34,35,36,88,89,90,91]

**Table 2:** Quantitative efficacy and risk matrix of tested chemical, biorational, and biological control agents against *Corythucha arcuata*.

Control Category	Active Agent/Strain	Target Stage	Quantitative Efficacy	Potential Risks and Limitations	References
Synthetic Chemical	Neonicotinoids and Pyrethroids	Adults, Nymphs	>90% mortality within 48 h	High non-target toxicity; risk of pest resistance; restricted in forests.	[104]
Biorational Chemical	Spinosad	Adults, Nymphs	85–95% mortality	Moderate risk to aquatic organisms; high application cost over large scales.	[75]
Biorational Chemical	Plant-derived compounds and IGRs	Nymphs	70–85% nymphal reduction	Variable field stability; requires precise phenological timing.	[46]
Biological (Fungi)	Beauveria pseudobassiana	Overwintering Adults	60–80% laboratory mortality	Dependent on ambient micro-relative humidity (>70%) for spore germination.	[56,57]
Biological (Parasitoid)	Erythmelus klopomor	Eggs (Oophagous)	Up to 75% egg parasitism	Requires complex multi-stage quarantine and environmental safety approvals.	[58,106]

**Table 3:** Comparative matrix of Chemical/Biorational Control versus Biological Control within an Integrated Pest Management (IPM) framework for *Corythucha arcuata*.

Evaluation Criteria	Chemical and Biorational Interventions	Classical and Augmentative Biological Control
Speed of Action	High: Provides immediate short-term knockdown and mortality of active populations.	Slow to Moderate: Requires time for fungal propagation or parasitoid population establishment.
Long-Term Durability	Low: Requires repeated seasonal applications; poses a continuous risk of pest resistance.	Moderate to High: Offers sustainable self-regulating dynamics once fully established, though initial establishment can be uncertain.
Ecological Selectivity	Low to Moderate: Synthetic compounds frequently harm non-target beneficial entomofauna.	High: Specialist agents like *E. klopomor* exhibit high host fidelity with minimal native risks.
Social and Public Acceptance	Low: Strongly opposed by citizens when applied near urban agglomerations or inhabited areas.	High: Well-accepted by professional foresters and local communities for long-term conservation.
Economic Cost-Efficiency	Low over time: Involves high, recurring costs for ground/aerial application protocols and chemical products.	High long-term potential: Economically sustainable post-release, but requires substantial initial investment for quarantine screening and rearing

## Data Availability

No new data were created or analyzed in this study. Data sharing is not applicable to this article.
